# The effect of denosumab on disseminated tumor cells (DTCs) of breast cancer patients with neoadjuvant treatment: a GeparX translational substudy

**DOI:** 10.1186/s13058-023-01619-2

**Published:** 2023-03-28

**Authors:** Pauline Wimberger, Jens-Uwe Blohmer, Petra Krabisch, Theresa Link, Marianne Just, Bruno Valentin Sinn, Eike Simon, Christine Solbach, Tanja Fehm, Carsten Denkert, Cristin Kühn, Kerstin Rhiem, Hans Tesch, Sherko Kümmel, Andrea Petzold, Oliver Stötzer, Cornelia Meisel, Jan Dominik Kuhlmann, Valentina Nekljudova, Sibylle Loibl

**Affiliations:** 1 Department of Gynecology and Obstetrics, Medical Faculty and University Hospital Carl Gustav Carus, Technische Universität Dresden, Fetscherstraße 74, 01307 Dresden, Germany; 2 National Center for Tumor Diseases (NCT), Dresden, Germany: German Cancer Research Center (DKFZ), Heidelberg, Germany; Faculty of Medicine and University Hospital Carl Gustav Carus, Technische Universität Dresden, Dresden, Germany; Helmholtz-Zentrum Dresden-Rossendorf (HZDR), Dresden, Germany; 3 German Cancer Consortium (DKTK), Dresden and German Cancer Research Center (DKFZ), Heidelberg, Germany; 4 Gynäkologie mit Brustzentrum, Charité-Univesitätsmedizin Berlin, Berlin, Germany; 5 Klinikum Chemnitz, Chemnitz, Germany; 6 Onkologische Schwerpunktpraxis Bielefeld, Bielefeld, Germany; 7 Department of Pathology, Charité – Universitätsmedizin Berlin, corporate member of Freie Universität Berlin and Humboldt Universität zu Berlin, Berlin, Germany; 8 Kreiskrankenhaus Torgau, Torgau, Germany; 9 Universitätsklinik Frankfurt, Frankfurt, Germany; 10 Universität Düsseldorf, Düsseldorf, Germany; 11 Institut für Pathologie, Philipps Universität Marburg und Universitätsklinikum Marburg (UKGM), Marburg, Germany; 12 Katharinen-Hospital Unna, Unna, Germany; 13 Universität Köln, Zentrum Familiärer Brust- und Eierstockkrebs, Köln, Germany; 14 Centrum für Hämatologie und Onkologie Bethanien, Frankfurt, Germany; 15 Kliniken Essen-Mitte Evang. Huyssen-Stiftung, Essen, Germany; 16 Gemeinschaftspraxis Hämatologie/Intern. Onkologie, München, Germany; 17 German Breast Group, Neu-Isenburg, Germany

**Keywords:** GeparX trial, Denosumab, Disseminated tumor cells, Bone marrow, Neoadjuvant chemotherapy

## Abstract

**Background:**

Disseminated tumor cells (DTCs) in the bone marrow are observed in about 40% at primary diagnosis of breast cancer and predict poor survival. While anti-resorptive therapy with bisphosphonates was shown to eradicate minimal residue disease in the bone marrow, the effect of denosumab on DTCs, particularly in the neoadjuvant setting, is largely unknown. The recent GeparX clinical trial reported that denosumab, applied as an add-on treatment to nab-paclitaxel based neoadjuvant chemotherapy (NACT), did not improve the patient’s pathologic complete response (pCR) rate. Herein, we analyzed the predictive value of DTCs for the response to NACT and interrogated whether neoadjuvant denosumab treatment may eradicate DTCs in the bone marrow.

**Methods:**

A total of 167 patients from the GeparX trial were analyzed for DTCs at baseline by immunocytochemistry using the pan-cytokeratin antibody A45-B/B3. Initially DTC-positive patients were re-analyzed for DTCs after NACT ± denosumab.

**Results:**

At baseline, DTCs were observed in 43/167 patients (25.7%) in the total cohort, however their presence did not predict response to nab-paclitaxel based NACT (pCR rates: 37.1% in DTC-negative vs. 32.6% DTC-positive; *p* = 0.713). Regarding breast cancer subtypes, the presence of DTCs at baseline was numerically associated with response to NACT in TNBC patients (pCR rates: 40.0% in DTC-positive vs. 66.7% in DTC-negative patients; *p* = 0.16). Overall, denosumab treatment did not significantly increase the given DTC-eradication rate of NACT (NACT: 69.6% DTC-eradication vs. NACT + denosumab: 77.8% DTC-eradication; *p* = 0.726). In TNBC patients with pCR, a numerical but statistically non-significant increase of DTC-eradication after NACT + denosumab was observed (NACT: 75% DTC-eradication vs. NACT + denosumab: 100% DTC-eradication; *p* = 1.00).

**Conclusion:**

This is the first study worldwide, demonstrating that neoadjuvant add-on denosumab over a short-term period of 24 months does not increase the DTC-eradication rate in breast cancer patients treated with NACT.

**Supplementary Information:**

The online version contains supplementary material available at 10.1186/s13058-023-01619-2.

## Introduction

Despite recent advances in early detection and systemic treatment, about 20–30% of patients with early breast cancer experience distant metastatic relapse. Recurrent disease can occur even years after primary treatment and constitutes the predominant cause of breast cancer specific death [1–4]. This is probably due to minimal residue disease, shaped by occult micrometastases, which have been seeded by early hematogenic dissemination [3, 5, 6]. Already at primary diagnosis of breast cancer, about 30–40% of patients have disseminated tumor cells (DTCs) in the bone marrow (BM) [7, 8]. It has been widely accepted that the presence of DTCs at primary diagnosis as well as their persistence after neoadjuvant chemotherapy (NACT) are both predictors of poor survival [7, 9–11].

Anti-resorptive agents, such as bisphosphonates, counteract osteoclast mediated bone-resorption and are widely used to treat patients, which suffer from bone metastasis induced skeletal adverse events or cancer treatment-induced bone loss and osteoporosis [12–15]. It is known that adjuvant bisphosphonates reduce the rate of breast cancer recurrence and improve prognosis in postmenopausal breast cancer patients [16, 17]. Moreover, oral ibandronate treatment of apparently disease-free patients was shown to completely eradicate persisting DTCs after 6–12 months [18], suggesting a direct effect of bisphosphates on micrometastasis in the bone marrow.

The human monoclonal IgG2 antibody denosumab represents a further class of anti-resorptive agents and targets receptor activator of nuclear factor-kappaB ligand (RANKL), [19]. Inactivation of RANKL by denosumab prevents RANKL signalling, which in turn reduces osteoclastic bone-resorption [20]. Comparable to bisphosphonates, denosumab is a well-established therapeutic option in breast cancer patients for the treatment of skeletal adverse events in metastatic bone disease, treatment-induced bone loss and osteoporosis [21, 22]. Moreover, RANK signaling was shown to contribute to the initiation and progression of breast cancer [23, 24]. Accordingly, RANKL and its receptor are highly expressed in breast cancer patients and predict poor prognosis [24–27].

Although there is a great body of pre-clinical evidence that RANK signaling promotes proliferation and (bone) metastatic progression of breast cancer [23, 28, 29], it still controversially discussed, whether targeted inhibition of RANK signalling by denosumab treatment will confer clinical benefit in patients with early breast cancer. While the ABCSG-18 trial showed that the addition of denosumab to adjuvant systemic treatment results in an improved disease-free survival [30], the D-CARE trial did not resolve any improvement of disease-related outcomes for high-risk early breast cancer patients, treated with denosumab [31]. Moreover, the phase IIb prospective randomized GeparX trial reported that denosumab, added to anthracycline/taxane-based NACT, did not improve pCR rates [32].

Serum RANKL levels were shown to be higher in DTC-positive compared to DTC-negative breast cancer patients and were reported to predict clinically manifest bone metastasis [33], suggesting a potential role of RANK-signaling in micrometastasis. However, whether denosumab eradicates DTCs, as it has been reported for bisphosphonates [18], is completely unknown. Using the framework of the recent GeparX study [32], we herein analyzed the clinical relevance of DTCs for predicting response to NACT and interrogated whether neoadjuvant denosumab treatment may eradicate DTCs in the bone marrow.

## Patients and methods

### Characterization of study patients and inclusion criteria

**Fig. 1:**
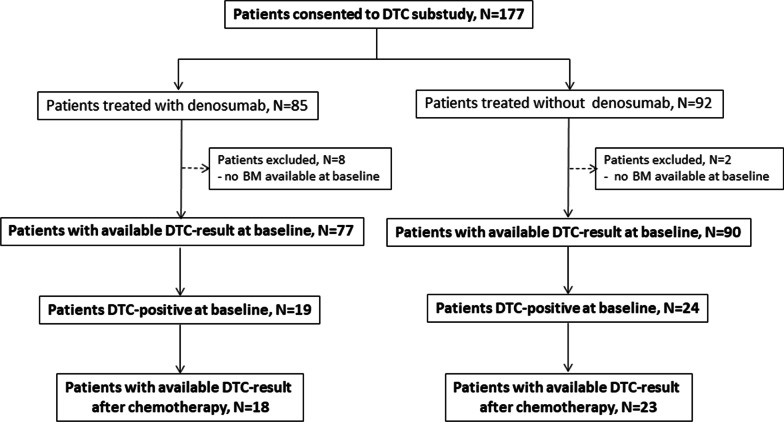
Conceptual workflow of the translational DTC substudy. The flow chart gives an overview on the inclusion of patients into the DTC substudy of the GeparX trial and the availability of DTC-results at baseline and after NACT. BM: bone marrow

The translational GeparX linked substudy was conducted at the Department of Gynecology and Obstetrics, University Hospital Carl Gustav Carus, TU Dresden, Germany. In total, 177 patients [32] were recruited from the GeparX trial and in 167/177 of these patients, bone marrow aspirates could be obtained (Fig. 1). The study was performed in accordance with good clinical practice guidelines, national laws and the Declaration of Helsinki. Informed written consent for DTC-analysis was obtained from all patients and the study was approved by the Local Research Ethics Committee (ethical vote number 2016315 and EK237082012).

### Collection and processing of bone marrow samples

Bone marrow samples were aspirated at baseline (before the beginning of neoadjuvant chemotherapy). In case of DTC-positivity, patients were subjected to a follow-up bone marrow aspiration during surgery. Isolation of the mononuclear cell (MNC) fraction from bone marrow was performed according to the recommendations for standardized tumor cell detection published by the German Consensus Group of Senology [34, 35]. Briefly, bone marrow was bilaterally aspirated from the anterior iliac crests (between 5–10 ml per site) under local anesthesia (or during surgery), heparinized (5000 U/ml) and processed within 24 h. MNCs were isolated from heparinized bone marrow (5000 U/ml) by Ficoll-Hypaque density gradient centrifugation (density 1.077 g/mol; Pharmacia, Freiburg, Germany) at 400× *g* for 30 min. Interphase cells were washed (400× *g* for 15 min) and re-suspended in phosphate buffered saline (PBS). A total of 1.5 × 10^6^ MNCs per area of 240 mm^2^ were directly spun onto glass slides (400× *g* for 5 min) coated with poly-L-lysine (Sigma, Deisenhofen, Germany) using a Hettich cytocentrifuge (Tuttlingen, Germany). In total, 9 × 10^6^ MNCs per patient were analyzed. The slides were air-dried overnight at room temperature.

### Immunocytochemistry

Immunocytochemical detection of cytokeratin (CK)-positive DTCs was performed, according to the recommendations for standardized tumor cell detection published by the German Consensus Group of Senology [34, 35]. Staining was performed using the murine monoclonal antibody A45-B/B3 (Micromet, Germany), directed against a common epitope of CK polypeptides including the CK heterodimers 8/18 and 8/19. The protocol has already been described in detail elsewhere [34, 35]. Briefly, the method includes (a) permeabilization of the cells with a detergent (5 min), (b) fixation with a formaldehyde-based solution (10 min), (c) binding of a A45-B/B3-alkaline phosphatase conjugate to cytoskeletal CKs (45 min) and (d) formation of an insoluble red reaction product at the site of binding of the CK-specific antibody conjugate (15 min) using the DAKO-APAAP detection kit (DakoCytomation, Denmark). All experimental steps were performed, according to the manufacturer’s instructions. Subsequently, the cells were mounted with Kaiser’s glycerol/gelatin (Merck, Darmstadt, Germany) in Tris–EDTA buffer (Sigma, Deisenhofen, Germany). A Fab-fragment-alkaline phosphatase conjugate (Micromet, Munich, Germany) served as negative control and did not show relevant background staining in human bone marrow samples. Furthermore, a positive control using the A45-B/B3-alkaline phosphatase conjugate and CK-expressing MCF-7 breast cancer cells (ATCC, Rockville, MD) was stained in parallel to each batch of patient samples under identical experimental conditions.

### Automated detection and classification of cytokeratin-positive DTCs

Microscopic evaluation of the CK-stained bone marrow samples for DTC-detection was carried out using the ARIOL system (Applied Imaging) according to the International Society for Haematotherapy and Graft Engineering (ISHAGE) evaluation criteria and the DTC consensus [34, 35]. This automated scanning microscope and imaging system consist of a slide loader, camera, computer and software. The software was specifically trained for the automated detection of CK-positive cells, based on particular colour, intensity, size, pattern, and shape. Each detected cell was reviewed and classified according to ISHAGE criteria by an experienced examinator. A patient was categorically considered DTC-positive, if at least one CK-positive cell was detectable in at least one of the two two-sided bone marrow aspirates.

### Statistical methods

Data analysis was performed using SAS^®^ (Statistical Analysis Software; version 9.4 under SAS Enterprise Guide 7.1 on Microsoft Windows 7 Enterprise). DTC presence at baseline, DTC-eradication after NACT and pCR rates (stratified by baseline DTCs or by DTC-eradication) were presented as descriptive bar charts. Groupwise comparisons were performed using the Fisher's Exact Test.

## Results

### The presence of DTCs at baseline and their eradication after NACT ± denosumab

**Fig. 2:**
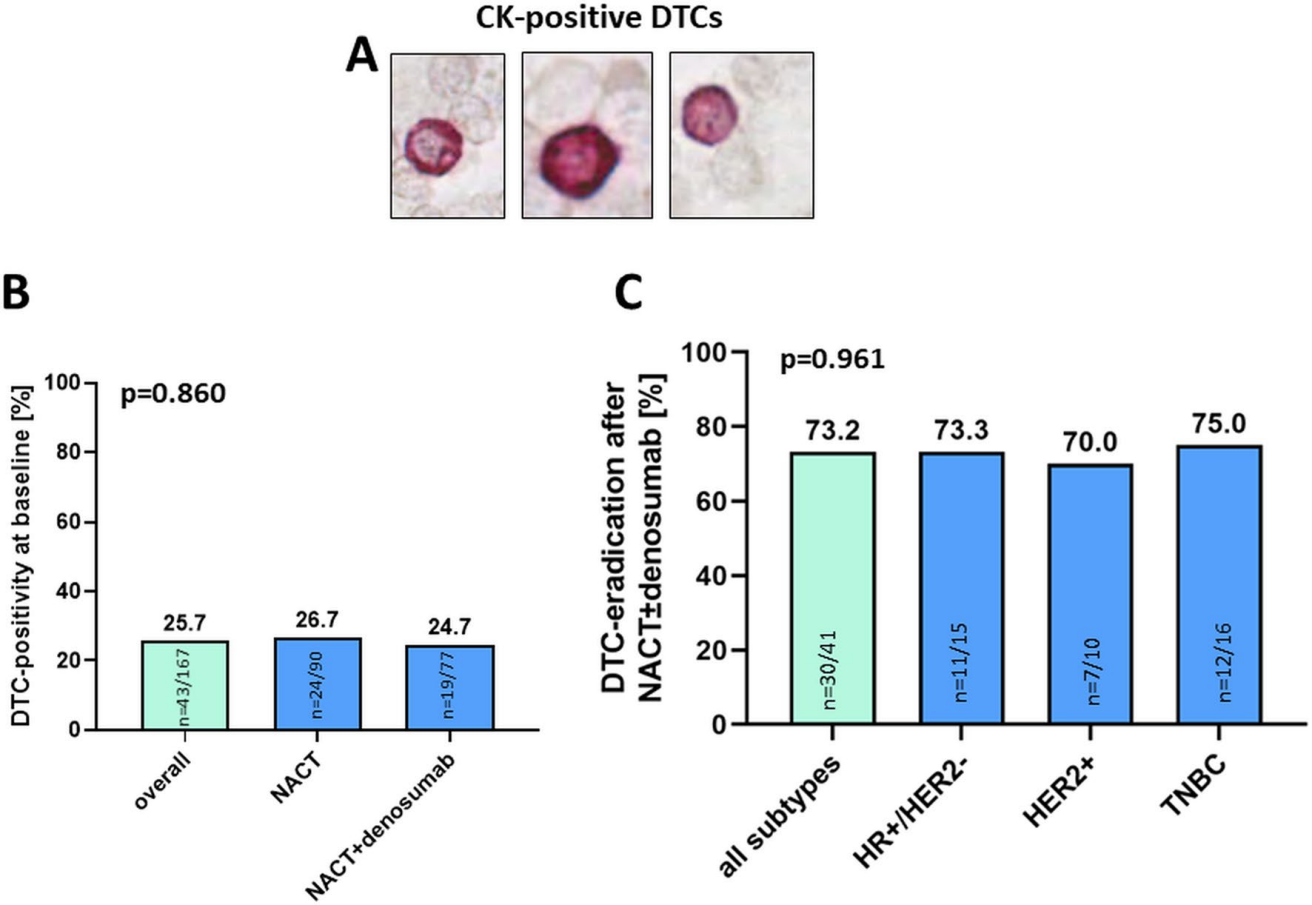
DTC-positivity at baseline and DTC-eradication after NACT ± denosumab. **A** Representative images of CK-positive DTCs in the bone marrow, stained by immunocytochemistry with the antibody A45-B/B3. **B** The bar chart shows the percentage of patients, being positive for DTCs in the bone marrow among the total cohort of the substudy and in the different study arm, i.e. in patients with NACT only and in patients with NACT + denosumab. **C** Bar charts showing the percentage of DTC-eradication in the bone marrow among the total cohort of the substudy and the different subtypes of breast cancer. *P* values according to the Fisher's Exact Test are indicated. HR: hormone receptor; HER2: human epidermal growth receptor 2; TNBC: triple-negative breast cancer

A total of 167 patients from the GeparX clinical trial were available for DTC-analysis (Fig. 1). Patient characteristics are shown in Supplementary Table 1. At baseline, the overall DTC-positivity was 25.7% (43/167 patients) with a median of 1 DTC per patient (range 1–9 DTCs per patient, Fig. 2A). The distribution of baseline parameters with regard to DTC-positivity is shown in Supplementary Table 2. Notably, DTC-positivity at baseline did not significantly differ in NACT vs. NACT + denosumab treated patients (26.7% vs. 24.7%; *p* = 0.860; Fig. 2B).

To monitor the rate of DTC-eradiation in response to NACT ± denosumab, we subsequently performed bone marrow re-puncture and DTC follow-up analysis in patients with a DTC-positive status at baseline. A total of 41/43 DTC-positive patients were available for this purpose. A patient was considered “DTC-eradicated”, if DTCs were initially present at baseline, followed by a negative DTC-status after NACT ± denosumab. We observed a high rate of DTC-eradication among baseline DTC-positive breast cancer patients after NACT ± denosumab (73.2%), which was consistent across the different subtypes of breast cancer (73.3% in HR + /HER2-; 70.0% in HER2 + ; 75.0% in TNBC; *p* = 0.961; Fig. 2C).

To sum up, we report a high rate of DTC-eradication (> 70%) in the total study population after NACT ± denosumab among baseline DTC-positive breast cancer patients.

### Predictive value of DTCs for response to nab-paclitaxel based NACT

A total of 60/167 (35.9%) patients had a pCR. Of those, HR+/HER2− patients had the lowest pCR rate (15.3%), followed by HER2+ patients (43.3%). The highest pCR rate was observed in TNBC patients (55.4%).

**Fig. 3:**
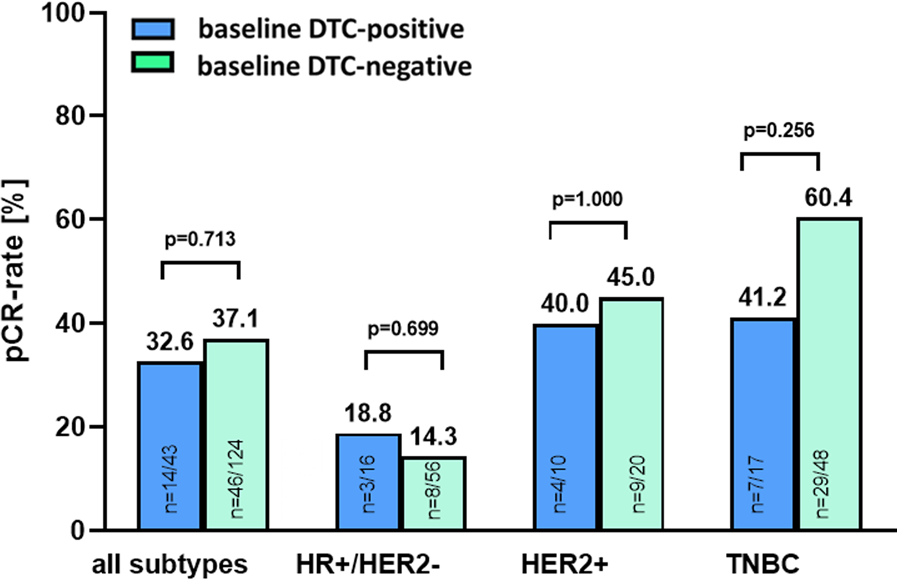
Predictive value of baseline DTCs. Bar chart showing pCR rate after NACT ± denosumab among the total cohort and the different subtypes of breast cancer with regard to DTC-status at baseline. *P* values according to the Fisher's Exact Test are indicated. HR: hormone receptor; HER2: human epidermal growth receptor 2; TNBC: triple-negative breast cancer

To investigate the predictive value of DTCs for response to NACT, we compared baseline DTC-status with the patient’s pCR rate after NACT ± denosumab. Overall, the pCR rate in DTC-positive patients was 32.6% versus 37.1% in DTC-negative patients (*p* = 0.713; Fig. 3). Thus, no statistically significant association between pCR rate and baseline DTC-positivity was reported. The same result was evident for HR+/HER2− and HER2+ subtypes, in which also no significant differences between the pCR rates in DTC-positive vs. DTC-negative patients were observed (HR+/HER2−: 18.8% vs. 14.3%, *p* = 0.699; HER2+: 40% vs. 45%, *p* = 1.000). Interestingly, TNBC patients with DTC-positivity at baseline had a numerically lower pCR rate than patients without evidence of DTCs (41.2% vs. 60.4%; 19.2% difference in pCR; *p* = 0.256, Fig. 3). Due to the limited number of patients, a further stratification regarding the NACT vs. NACT + denosumab arm was not reasonable at this point.

**Fig. 4:**
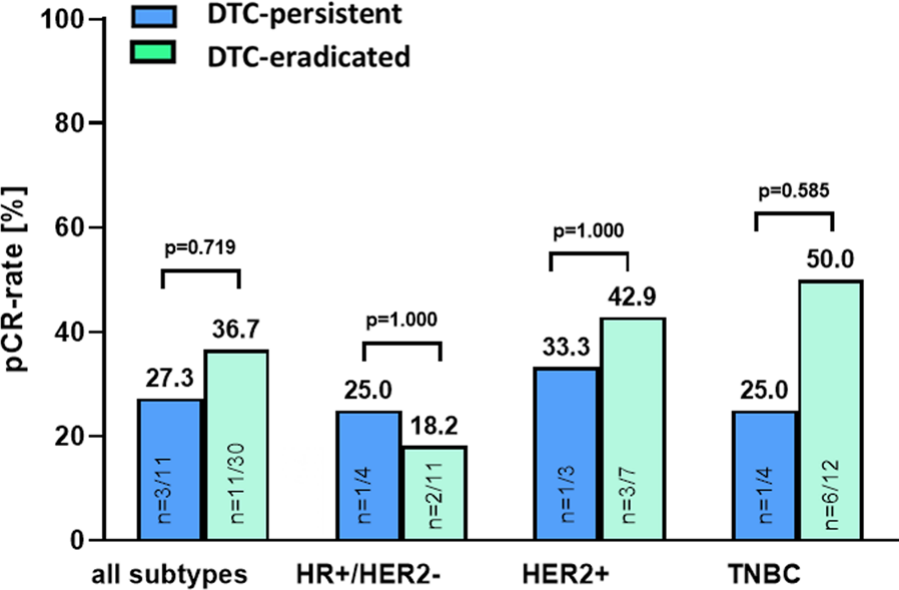
Association between pCR rate and DTC-eradication. Bar chart showing pCR rate after NACT ± denosumab among the total cohort and the different subtypes of breast cancer with regard to DTC-eradication. *P* values according to the Fisher's Exact Test are indicated. HR: hormone receptor; HER2: human epidermal growth receptor 2; TNBC: triple-negative breast cancer

We further analyzed, whether a pCR to NACT ± denosumab may parallel DTC-eradication in the bone marrow. Across all subtypes, there was no statistical significance between pCR rate and DTC-eradication. Thus, the pCR rate in DTC-persistent patients was 27.3% versus 36.7% in DTC-eradicated patients (*p* = 0.719; Fig. 4). The same trend was observed for HR+/HER2− and HER2+ subtypes, in which no significant differences between the pCR rates in DTC-persistent vs. DTC-eradicated patients were observed (25.0% vs. 18.2% in HR+ /HER2− , *p* = 1.000; 33.3% vs. 42.9% in HER2+ , *p* = 1.000). Again, in patients with TNBC, there was a numerical trend towards a possible association between DTC-persistence and decreased pCR rate (25.0% vs. 50.0%; 25% difference in pCR; *p* = 0.585, Fig. 4). Due to the limited number of patients, a further stratification regarding the NACT versus NACT + denosumab arm was not reasonable at this point.

We conclude that the presence of DTCs at baseline does not predict overall response to NACT ± denosumab. Moreover, there was no association between pCR and DTC-eradication. However, subtype analysis showed some numerical trends for a possible association between DTCs and pCR in TNBC.

### The effect of denosumab on DTCs

**Fig. 5:**
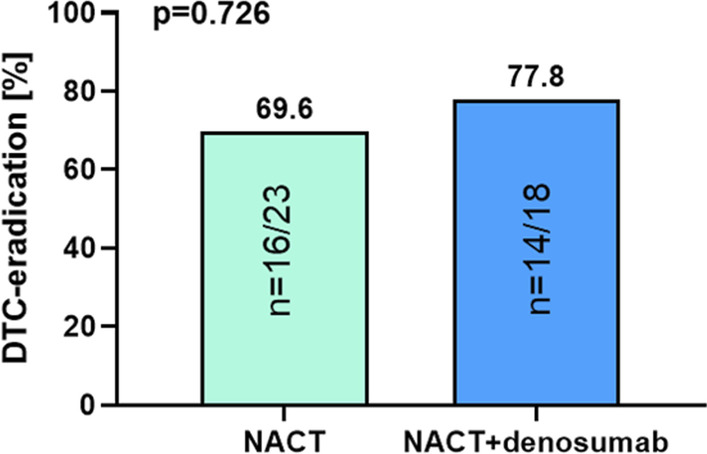
Effect of denosumab on DTC-eradication. Bar chart showing DTC-eradication rates in patients with NACT vs. patients with NACT + denosumab. *P* values according to the Fisher's Exact Test are indicated

We inquired, whether neoadjuvant add-on denosumab treatment, framed by the GeparX study [32], may eradicate DTCs in the bone marrow. Therefore, we analyzed, whether denosumab may increase the given DTC-eradication rate by NACT. DTC-eradication in the NACT + denosumab arm (77.8%) was numerically higher compared to the NACT arm without denosumab (69.6%). However, this difference did not reach statistical significance (*p* = 0.726; Fig. 5). In TNBC patients with a pCR, 0/3 patients (0%) were DTC-positive after NACT + denosumab (*p* = 0.429), whereas 1/4 patients (25%) were DTC-positive after NACT alone (*p* = 1.00).

Taken together, denosumab did not increase the overall DTC-eradication rate by NACT. However, subtype analysis shows a numerical trend towards a possible effect of denosumab on DTCs in TNBC.

## Discussion

This is the first study worldwide, analyzing the effect of neoadjuvant denosumab on breast cancer DTCs. Overall DTC-positivity in our study cohort (25.7%) was comparable to that reported in a comprehensive meta-analysis of 4703 patients with stage I-III breast cancer (30.6%) [7], excluding any bias in our study towards methodology and patient selection. Moreover, DTC-positivity at baseline was well balanced between the denosumab and non-denosumab treated study arm, indicating that there is no additional selection bias between the different study arms, which may have confounded our results.

The fact that (1) anti-resorptive therapy with bisphosphonates is already known to eradicate DTCs in the BM [18] and that (2) circulating levels of serum RANKL are elevated in DTC-positive breast cancer patients [33], provided a strong rationale for us to hypothesize that anti-resorptive denosumab likewise promotes the eradication of DTCs in the BM. Therefore, we re-analyzed patients with baseline DTC-positivity for the presence of DTCs after NACT ± denosumab, in order to distinguish between DTC-eradication vs. DTC-persistence. We observed a substantial DTC-eradication rate after NACT ± denosumab (73%). So far, only little is known about the effectiveness of NACT in eradicating DTCs in the BM, since previous reports primarily focused on the prognostic significance of DTCs after NACT [9–11]. In a previous study on breast cancer, DTC-positivity in the overall study population (adjuvant and neoadjuvant treatment) was 29%. Interestingly, in those patients with neoadjuvant treatment, DTC-positivity after NACT was still 25%, suggesting that overall DTC-positivity was not substantially decreased by NACT [36]. In the adjuvant setting, inconclusive results with regard to DTC-eradication after treatment have been reported [37–41]. Those opposing results could likely be due to use to confounding biases, with regard to therapy regimes, patient selection or different DTC-detection methods. Moreover, our study is not directly comparable to others, since we conceptually assessed the DTC-eradication rate by follow-up analysis of a pre-selected cohort of 100% DTC-baseline positive patients, so that “negative to positive switchers”, which also influence the overall DTC-frequency after NACT, could not be considered.

We report for the overall cohort that (1) DTC-positivity at baseline is non-predictive for response to NACT and that (2) DTC-eradication does not parallel the pCR rate. These finding are supported by a previous study to show that there is no overall association between pCR and DTC-status after NACT [10]. It could be hypothesized that DTCs undergo an independent metastatic progression in parallel to the primary tumor [42], so that their chemosensitivity could be different to that of the primary tumor mass. This may explain that DTCs in our study neither predicted response to NACT nor their eradication reflected a pCR. Nevertheless, we observed a numerical trend towards a predictive value of DTCs in TNBC. This could be of high clinical interest and requires further investigation, since the pCR rate is higher and the association between pCR and outcome is more pronounced in TNBC compared to the HR+/HER2− subtype [43].

The underlying GeparX trial showed that the addition of denosumab to NACT did not increase pCR [32]. In line with these findings, we could not observe an overall effect of denosumab on DTCs in the GeparX study cohort. However, this result refers to short-term denosumab treatment (24 months, 6 applications), as it was framed by the GeparX trial design [32]. Long-term follow-up data of the GeparX study will be awaited in the next years, in order to analyze the long-term effect of denosumab on DTCs and on the patient’s survival. Nonetheless, we observed again in TNBC patients, that there was a numerical trend towards an increase of DTC-eradication by denosumab. This trend is in line with the previous observation, that TNBC, in comparison to the other intrinsic subtypes, is generally more likely to show DTC-eradication after NACT [36]. We hypothesize that DTCs of TNBC could possibly be more sensitive to anti-resorptive therapy with denosumab, since RANK is overexpressed in this breast cancer subtype [44]. Moreover, RANKL, which is expressed in response to progesterone in progesterone receptor (PR)-positive luminal epithelial cells, has a paracrine proliferative effect on neighboring PR-negative basal cells [24], suggesting a potential dependency of basal-like cancer, which is enriched in TNBC [45], to RANK signaling. Due to the limited number of TNBC patients, our statistical analysis was of limited information value, however, our results encourage further investigation of the denosumab effect on DTCs in TNBC patients. Considering the prognostic impact and the potentially (dormant) stem-like state of persisting DTCs after NACT [9–11, 46], further studies should address, whether denosumab could possibly be used as a cell cycle-independent drug for DTC-eradication in TNBC.

## Conclusion

This is the first study worldwide, demonstrating that neoadjuvant add-on denosumab over a short-term period of 24 months does not increase the DTC-eradication rate in breast cancer patients treated with NACT. Nevertheless, our results suggest a trend towards a potential predictive effect of DTCs and an increased DTC-eradication by denosumab in TNBC, which warrants further investigation.

## Supplementary Information

**Additional file 1: Table S1.** Baseline Characteristics of the DTC substudy.

**Additional file 2: Table S2.** Baseline Characteristics in DTC-positive vs. DTC-negative patients

## Data Availability

All data generated or analysed during this study are included in this published article.

## Abbreviations

DTCs: Disseminated tumor cells
BC: Breast cancer
NACT: Neoadjuvant chemotherapy
pts: Patients
TNBC: Triple-negative breast cancer
pCR: Pathologic complete response
BM: Bone marrow
RANKL: Receptor activator of NFĸB
RANKL: Receptor activator of NFĸB ligand
PBS: Phosphate buffered saline
MNCs: Mononuclear cells
ISHAGE: International Society for Hematotherapy and Graft Engineering
PR: Progesterone receptor
