# Supplementary material for: The effect of denosumab on disseminated tumor cells (DTCs) of breast cancer patients with neoadjuvant treatment: a GeparX translational substudy
Source: Breast Cancer Res. 2023 Mar 28;25:32. doi: 10.1186/s13058-023-01619-2 (PMC10045108; doi:10.1186/s13058-023-01619-2)
Supplement: Supplementary file 1 — Additional file 1: Table S1. Baseline Characteristics of the DTC substudy. [file 13058_2023_1619_MOESM1_ESM.docx]

**Supplementary Table 1: Baseline Characteristics of the DTC substudy**

| *Parameter* | *Parameter value* | *With Denosumab* | *Without Denosumab* | *Overall* | *p-value* |
| --- | --- | --- | --- | --- | --- |
| Age, years | <30 | 1 ( 1.3) | 2 ( 2.2) | 3 ( 1.8) | 0.537 |
|  | 30-<40 | 14 (18.2) | 14 (15.6) | 28 (16.8) |  |
|  | 40-<50 | 27 (35.1) | 33 (36.7) | 60 (35.9) |  |
|  | 50-<60 | 18 (23.4) | 23 (25.6) | 41 (24.6) |  |
|  | 60-<70 | 12 (15.6) | 17 (18.9) | 29 (17.4) |  |
|  | 70+ | 5 ( 6.5) | 1 ( 1.1) | 6 ( 3.6) |  |
|  | missing | 0 | 0 | 0 |  |
| Gender | female | 76 (98.7) | 90 ( 100) | 166 (99.4) | 0.461 |
|  | male | 1 ( 1.3) | 0 ( 0.0) | 1 ( 0.6) |  |
|  | missing | 0 | 0 | 0 |  |
| Menopausal status | premenopausal | 42 (54.5) | 55 (61.1) | 97 (58.1) | 0.416 |
|  | postmenopausal | 34 (44.2) | 35 (38.9) | 69 (41.3) |  |
|  | n.a. (male patient) | 1 ( 1.3) | 0 ( 0.0) | 1 ( 0.6) |  |
|  | missing | 0 | 0 | 0 |  |
| Karnofsky index | 80% | 0 ( 0.0) | 0 ( 0.0) | 0 ( 0.0) | 0.654 |
|  | 90% | 1 ( 1.3) | 2 ( 2.2) | 3 ( 1.8) |  |
|  | 100% | 76 (98.7) | 88 (97.8) | 164 (98.2) |  |
|  | missing | 0 | 0 | 0 |  |
| Tumor site | unilateral right | 36 (46.8) | 47 (52.2) | 83 (49.7) | 0.437 |
|  | unilateral left | 38 (49.4) | 42 (46.7) | 80 (47.9) |  |
|  | bilateral | 3 ( 3.9) | 1 ( 1.1) | 4 ( 2.4) |  |
|  | missing | 0 | 0 | 0 |  |
| Tumor focality, by palpation | unifocal | 65 (84.4) | 80 (88.9) | 145 (86.8) | 0.299 |
|  | multifocal | 8 (10.4) | 9 (10.0) | 17 (10.2) |  |
|  | multicentric | 4 ( 5.2) | 1 ( 1.1) | 5 ( 3.0) |  |
|  | missing | 0 | 0 | 0 |  |
| Tumor focality, by sonogrpahy | unifocal | 57 (74.0) | 76 (84.4) | 133 (79.6) | 0.188 |
|  | multifocal | 15 (19.5) | 12 (13.3) | 27 (16.2) |  |
|  | multicentric | 5 ( 6.5) | 2 ( 2.2) | 7 ( 4.2) |  |
|  | missing | 0 | 0 | 0 |  |
| cT by palpation | cT1 | 16 (22.2) | 33 (40.7) | 49 (32.0) | 0.013 |
|  | cT2 | 47 (65.3) | 41 (50.6) | 88 (57.5) |  |
|  | cT3 | 9 (12.5) | 4 ( 4.9) | 13 ( 8.5) |  |
|  | cT4 | 0 ( 0.0) | 3 ( 3.7) | 3 ( 2.0) |  |
|  | missing | 5 | 9 | 14 |  |
| cT by sonography | cT1 | 21 (27.3) | 39 (43.3) | 60 (35.9) | 0.044 |
|  | cT2 | 54 (70.1) | 46 (51.1) | 100 (59.9) |  |
|  | cT3 | 2 ( 2.6) | 2 ( 2.2) | 4 ( 2.4) |  |
|  | cT4 | 0 ( 0.0) | 3 ( 3.3) | 3 ( 1.8) |  |
|  | missing | 0 | 0 | 0 |  |
| cN by palpation | cN0 | 49 (63.6) | 58 (65.2) | 107 (64.5) | 0.200 |
|  | cN1 | 19 (24.7) | 27 (30.3) | 46 (27.7) |  |
|  | cN2 | 9 (11.7) | 4 ( 4.5) | 13 ( 7.8) |  |
|  | cN3 | 0 ( 0.0) | 0 ( 0.0) | 0 ( 0.0) |  |
|  | missing | 0 | 1 | 1 |  |
| cN by sonography | cN0 | 42 (54.5) | 53 (58.9) | 95 (56.9) | 0.382 |
|  | cN1 | 25 (32.5) | 32 (35.6) | 57 (34.1) |  |
|  | cN2 | 9 (11.7) | 4 ( 4.4) | 13 ( 7.8) |  |
|  | cN3 | 1 ( 1.3) | 1 ( 1.1) | 2 ( 1.2) |  |
|  | missing | 0 | 0 | 0 |  |
| Sentinel node biopsy (not recommended) | none | 58 (75.3) | 70 (77.8) | 128 (76.6) | 0.759 |
|  | negative | 15 (19.5) | 14 (15.6) | 29 (17.4) |  |
|  | positive | 4 ( 5.2) | 6 ( 6.7) | 10 ( 6.0) |  |
|  | no sentinel detected | 0 ( 0.0) | 0 ( 0.0) | 0 ( 0.0) |  |
|  | missing | 0 | 0 | 0 |  |
| Core- or fine needle biopsy of lymph node | none | 64 (83.1) | 84 (93.3) | 148 (88.6) | 0.057 |
|  | negative | 3 ( 3.9) | 0 ( 0.0) | 3 ( 1.8) |  |
|  | positive | 10 (13.0) | 6 ( 6.7) | 16 ( 9.6) |  |
|  | missing | 0 | 0 | 0 |  |
| cN combined* | cN0 | 44 (57.1) | 52 (57.8) | 96 (57.5) | 1.000 |
|  | cN+ | 33 (42.9) | 38 (42.2) | 71 (42.5) |  |
|  | missing | 0 | 0 | 0 |  |
| ER/PgR, local assessment | both ER and PgR negative | 27 (35.1) | 41 (45.6) | 68 (40.7) | 0.207 |
|  | ER and/or PgR positive | 50 (64.9) | 49 (54.4) | 99 (59.3) |  |
|  | missing | 0 | 0 | 0 |  |
| ER/PgR, central pathology | both ER and PgR negative | 29 (37.7) | 44 (48.9) | 73 (43.7) | 0.161 |
|  | ER and/or PgR positive | 48 (62.3) | 46 (51.1) | 94 (56.3) |  |
|  | missing | 0 | 0 | 0 |  |
| HER2 status (loc. pathology) | positive | 17 (22.1) | 18 (20.0) | 35 (21.0) | 0.849 |
|  | negative | 60 (77.9) | 72 (80.0) | 132 (79.0) |  |
|  | missing | 0 | 0 | 0 |  |
| HER2 status (centr. pathology) | positive | 16 (20.8) | 14 (15.6) | 30 (18.0) | 0.423 |
|  | negative | 61 (79.2) | 76 (84.4) | 137 (82.0) |  |
|  | missing | 0 | 0 | 0 |  |
| Breast cancer subtype (stratification) | HER2-/HR+ | 36 (46.8) | 36 (40.0) | 72 (43.1) | 0.273 |
|  | TNBC | 25 (32.5) | 40 (44.4) | 65 (38.9) |  |
|  | HER2+ | 16 (20.8) | 14 (15.6) | 30 (18.0) |  |
|  | missing | 0 | 0 | 0 |  |
| Tumor grading | G1 | 2 ( 2.6) | 2 ( 2.2) | 4 ( 2.4) | 0.491 |
|  | G2 | 35 (45.5) | 33 (36.7) | 68 (40.7) |  |
|  | G3 | 40 (51.9) | 55 (61.1) | 95 (56.9) |  |
|  | missing | 0 | 0 | 0 |  |
| Histological tumor type | Invasive carcinoma of no special type (NST) | 76 (98.7) | 87 (96.7) | 163 (97.6) | 0.419 |
|  | Invasive lobular carcinoma or mixed lobular carcinoma | 1 ( 1.3) | 1 ( 1.1) | 2 ( 1.2) |  |
|  | other | 0 ( 0.0) | 2 ( 2.2) | 2 ( 1.2) |  |
|  | missing | 0 | 0 | 0 |  |
| Ki67, central pathology | <=20% | 16 (20.8) | 15 (16.7) | 31 (18.6) | 0.552 |
|  | >20% | 61 (79.2) | 75 (83.3) | 136 (81.4) |  |
|  | missing | 0 | 0 | 0 |  |
| LPBC (stratification) | no LPBC | 72 (93.5) | 83 (92.2) | 155 (92.8) | 1.000 |
|  | LPBC | 5 ( 6.5) | 7 ( 7.8) | 12 ( 7.2) |  |
|  | missing | 0 | 0 | 0 |  |
| Planned EC schedule (stratification) | 2-weekly | 60 (77.9) | 71 (78.9) | 131 (78.4) | 1.000 |
|  | 3-weekly | 17 (22.1) | 19 (21.1) | 36 (21.6) |  |
|  | missing | 0 | 0 | 0 |  |

*cN+ = positive lymph node by palpation and/or sonography and/or biopsy; cN0 = negative lymph node status by palpation and/or sonography and/or biopsy
