# Supplementary material for: The effect of denosumab on disseminated tumor cells (DTCs) of breast cancer patients with neoadjuvant treatment: a GeparX translational substudy
Source: Breast Cancer Res. 2023 Mar 28;25:32. doi: 10.1186/s13058-023-01619-2 (PMC10045108; doi:10.1186/s13058-023-01619-2)
Supplement: Supplementary file 2 — Additional file 2: Table S2. Baseline Characteristics in DTC-positive vs. DTC-negative patients [file 13058_2023_1619_MOESM2_ESM.docx]

**Supplementary Table 2: Baseline Characteristics in DTC-positive vs. DTC-negative patients**

| *Parameter* | *Parameter value* | *DTC negative at BL* | *DTC positive at BL* | *Overall* | *p-value* |
| --- | --- | --- | --- | --- | --- |
| Age, years | <30 | 1 ( 0.8) | 2 ( 4.7) | 3 ( 1.8) | 0.131 |
|  | 30-<40 | 22 (17.7) | 6 (14.0) | 28 (16.8) |  |
|  | 40-<50 | 45 (36.3) | 15 (34.9) | 60 (35.9) |  |
|  | 50-<60 | 34 (27.4) | 7 (16.3) | 41 (24.6) |  |
|  | 60-<70 | 17 (13.7) | 12 (27.9) | 29 (17.4) |  |
|  | 70+ | 5 ( 4.0) | 1 ( 2.3) | 6 ( 3.6) |  |
|  | missing | 0 | 0 | 0 |  |
| cT by palpation | cT1 | 41 (36.3) | 8 (20.0) | 49 (32.0) | 0.255 |
|  | cT2 | 60 (53.1) | 28 (70.0) | 88 (57.5) |  |
|  | cT3 | 10 ( 8.8) | 3 ( 7.5) | 13 ( 8.5) |  |
|  | cT4 | 2 ( 1.8) | 1 ( 2.5) | 3 ( 2.0) |  |
|  | missing | 11 | 3 | 14 |  |
| cT by sonography | cT1 | 46 (37.1) | 14 (32.6) | 60 (35.9) | 0.949 |
|  | cT2 | 73 (58.9) | 27 (62.8) | 100 (59.9) |  |
|  | cT3 | 3 ( 2.4) | 1 ( 2.3) | 4 ( 2.4) |  |
|  | cT4 | 2 ( 1.6) | 1 ( 2.3) | 3 ( 1.8) |  |
|  | missing | 0 | 0 | 0 |  |
| cN by palpation | cN0 | 81 (65.9) | 26 (60.5) | 107 (64.5) | 0.708 |
|  | cN1 | 32 (26.0) | 14 (32.6) | 46 (27.7) |  |
|  | cN2 | 10 ( 8.1) | 3 ( 7.0) | 13 ( 7.8) |  |
|  | cN3 | 0 ( 0.0) | 0 ( 0.0) | 0 ( 0.0) |  |
|  | missing | 1 | 0 | 1 |  |
| cN by sonography | cN0 | 73 (58.9) | 22 (51.2) | 95 (56.9) | 0.554 |
|  | cN1 | 39 (31.5) | 18 (41.9) | 57 (34.1) |  |
|  | cN2 | 10 ( 8.1) | 3 ( 7.0) | 13 ( 7.8) |  |
|  | cN3 | 2 ( 1.6) | 0 ( 0.0) | 2 ( 1.2) |  |
|  | missing | 0 | 0 | 0 |  |
| cN combined* | cN0 | 74 (59.7) | 22 (51.2) | 96 (57.5) | 0.373 |
|  | cN+ | 50 (40.3) | 21 (48.8) | 71 (42.5) |  |
|  | missing | 0 | 0 | 0 |  |
| Sentinel node biopsy (not recommended) | none | 93 (75.0) | 35 (81.4) | 128 (76.6) | 0.694 |
|  | negative | 23 (18.5) | 6 (14.0) | 29 (17.4) |  |
|  | positive | 8 ( 6.5) | 2 ( 4.7) | 10 ( 6.0) |  |
| Breast cancer subtype (stratification) | HER2-/HR+ | 56 (45.2) | 16 (37.2) | 72 (43.1) | 0.502 |
|  | TNBC | 48 (38.7) | 17 (39.5) | 65 (38.9) |  |
|  | HER2+ | 20 (16.1) | 10 (23.3) | 30 (18.0) |  |
|  | missing | 0 | 0 | 0 |  |
| Tumor grading | G1 | 2 ( 1.6) | 2 ( 4.7) | 4 ( 2.4) | 0.532 |
|  | G2 | 51 (41.1) | 17 (39.5) | 68 (40.7) |  |
|  | G3 | 71 (57.3) | 24 (55.8) | 95 (56.9) |  |
|  | missing | 0 | 0 | 0 |  |
| Histological tumor type | Invasive carcinoma of no special type (NST) | 122 (98.4) | 41 (95.3) | 163 (97.6) | **0.039** |
|  | Invasive lobular carcinoma or mixed lobular carcinoma | 0 ( 0.0) | 2 ( 4.7) | 2 ( 1.2) |  |
|  | other | 2 ( 1.6) | 0 ( 0.0) | 2 ( 1.2) |  |
|  | missing | 0 | 0 | 0 |  |
| Ki-67, central pathology (stratification) | <=20% | 23 (18.5) | 8 (18.6) | 31 (18.6) | 1.000 |
|  | >20% | 101 (81.5) | 35 (81.4) | 136 (81.4) |  |
|  | missing | 0 | 0 | 0 |  |

*cN+ = positive lymph node by palpation and/or sonography and/or biopsy; cN0 = negative lymph node status by palpation and/or sonography and/or biopsy
